# Chemistry of conjugation to gold nanoparticles affects G-protein activity differently

**DOI:** 10.1186/1477-3155-11-7

**Published:** 2013-03-19

**Authors:** Vibha Singh, Santhosh P Nagappan Nair, Gopala Krishna Aradhyam

**Affiliations:** 1 Department of Biotechnology, Indian Institute of Technology Madras, Chennai, 600036, India; 2 Department of Physics, Indian Institute of Technology Madras, Chennai, 600036, India

**Keywords:** G-protein, Nanoparticles, Fluorescence, Bioconjugation

## Abstract

**Background:**

Gold nanoparticles (AuNP) are extensively used as biophysical tools in the area of medicine and technology due to their distinct properties. However, vivid understanding of the consequences of biomolecule-nanomaterial interactions is still lacking. In this context, we explore the affect of conjugation of Gα_i1_ subunit (of heterotrimeric G-proteins) to AuNP and examine its consequences. We consider two bio-conjugation strategies covalent and non-covalent binding.

**Results:**

Affinity of the AuNP to the Gα_i1_ is 7.58 × 10 ^12^ M^-1^. AuNP conjugated Gα_i1_ exhibits altered kinetics of activation, non-covalent bio-conjugates displays retarded kinetics, up to 0.88 fold when GTPγS was used as ligand, of protein activation contrary to covalent conjugates which accelerates it to ~ 5 fold. Conjugation influence intrinsic Gαi1 GTPase function in conflicting modes. Non-covalent conjugation inhibits GTPase function (decrease in activity upto 0.8 fold) whilst covalent conjugation drastically accelerates it (12 fold increase in activity). Altered basal nucleotide uptake in both types of conjugates and GTPase function in non-covalent conjugate are almost comparable except for GTPase property of covalent conjugate. The effect is despite the fact that conjugation does not change global conformation of the protein.

**Conclusion:**

These findings provide clear evidence that nanoparticles, in addition to ‘passive interaction’ with protein (biomolecule), can interact “actively” with biomolecule and modify its function. This concept should be considered while engineering nanoparticle based delivery systems in medicine.

## Background

Impressive developments have occurred in nanoscience technology in the past decade, despite which a detailed understanding of nanoparticle (NP) interaction at cellular, sub-cellular and biomolecule level is lagging behind [1-19]. Cedervall *et al.* have demonstrated that binding and dissociation parameters of protein-nanoparticle complex depend on surface characteristics of nanoparticle as well as physico-chemical properties of the protein [20]. It has been demonstrated that NPs can elevate the rate of protein fibrillation potentially leading to proposals of novel mechanisms for amyloid diseases offering therapeutic opportunities for treatment [21]. Further, imaging studies provide crucial information that nano-conjugation uniformly promotes endocytosis of EGFR, influencing its compartmentalization, and the mechanism of endocytosis [22]. Thus, nano-conjugation cannot be construed as an innocuous tool but may directly alter the cellular processes at the molecular level [22]. Improved understanding of the interactions at nano-bio interface will give answers to questions concerning the effect of conjugation on protein conformation and hence its function.

Majority of the drugs target GPCR, which transduce signal by activating heterotrimeric G-protein which in turn switches on a cascade of downstream signal transduction pathway. The activation status of heterotrimeric G protein regulates the downstream cascade events. Hence, Gα_i1_ is a very important model protein to investigate the effects of different types of conjugation to nanoparticle. G proteins are ubiquitously expressed and despite the variety in their function and biochemical effects, their structures are very highly conserved. These properties of G proteins, additionally, make them very vital model systems for studying the effects of nanoparticles; an area that is fast gaining importance in biology and medicine.

In the present study, we investigate the effect of the different conjugation strategies on the conformation and function of G proteins. A comparative study is presented, between non-covalently and covalently bound AuNP-Gα_i1_ conjugates. In the non- covalent conjugate, the rate of basal nucleotide uptake was retarded in a concentration dependent manner of AuNP, whereas in the covalent conjugate, the rate was accelerated. Both types of conjugation influenced the intrinsic Gα_i1_ GTPase function affecting the kinetics of GTP hydrolysis in opposite modes. Non-covalent conjugation showed inhibitory effect on GTPase function whilst covalent conjugation dramatically accelerated it. We propose that the mode of interaction with nanoparticles modulate the function of the protein in the conjugate, which may alter related cellular physiological pathways. These findings provide strong evidence that nanoparticles can interact “actively” with biomolecules and modify their function.

## Results

### Bio-conjugation exploiting two different approaches

In this study two linkage strategies have been used for the conjugation of AuNPs to Gα_i1_. Dihydrolipoic acid (DHLA) capped AuNPs of hydrodynamic diameter ~ 6 nm was used in the entire study. Interaction studies were performed in a buffer at pH 8.0 with low ionic strength (10 mM NaCl), since conjugates exhibited a tendency to aggregate at higher ionic strength [23].

(i) *N-terminal covalent conjugation using EDC chemistry:* Site specific conjugation was achieved by forming a peptide bond between N-terminal primary amine of the protein and carboxylic acid groups of negatively charged AuNP utilizing 1-Ethyl-3-[3-dimethylaminopropyl] carbodiimide, EDC, chemistry. Retardation of AuNP’s electrophoretic mobility on agarose gel confirmed conjugation (Additional file 1: Figure S1A). Negative control (AuNP in presence of EDC without protein) also exhibited negligibly small amount of retardation in mobility due to the formation of O-acylisourea intermediate between AuNP and EDC. Changes in mobility of Au:Gα_i1_ complex depends on the concentration of EDC used.

(ii) *Non-covalent conjugation:* In non-covalent conjugation AuNP capping ligand plays an important role in the bio-conjugate. In the present study, AuNP is capped with DHLA which gives an overall negative charge on its surface. The protein may interact with AuNP in a number of orientations, or many AuNP’s could be attached to a given molecule of protein. Non-covalently bound protein-NP complex was also retarded in electrophoretic mobility, compared to AuNP itself (Additional file 1: Figure S1B), confirming their conjugation. To explore further, whether the conjugation was cysteine mediated, Gα_i1_ sulfhydryl groups were modified using Iodoacetamide. Conjugation to AuNP was observed even with cysteine modified Gα_i1_ (Additional file 1: Figure S1B), as evidenced by retardation in its electrophoretic mobility. Mobility of Gα_i1_-AuNP and cysteine-modified-Gα_i1_-AuNP were similar, ruling out cysteine mediated interaction between AuNP and Gα_i1_. To further demonstrate that non-specifically conjugated Gα_i1_ has free sulphydryl groups, N-(3-pyrene) maleimide (NPM) was used to check formation of fluorescent adducts with free thiol groups (Additional file 2: S2). Cysteine-modified Gα_i1_-AuNP, upon treatment with NPM displayed fluorescence spectrum with peaks at 377 nm, 397 nm and 418 nm similar to Gα_i1_-NPM adduct, though, with a lesser intensity. These results confirm the presence of free sulphydryl groups of Gα_i1_ even after non specific conjugation with AuNPs.

### Quenching of Gα_i1_ Tryptophan fluorescence by AuNP

**Figure 1:**
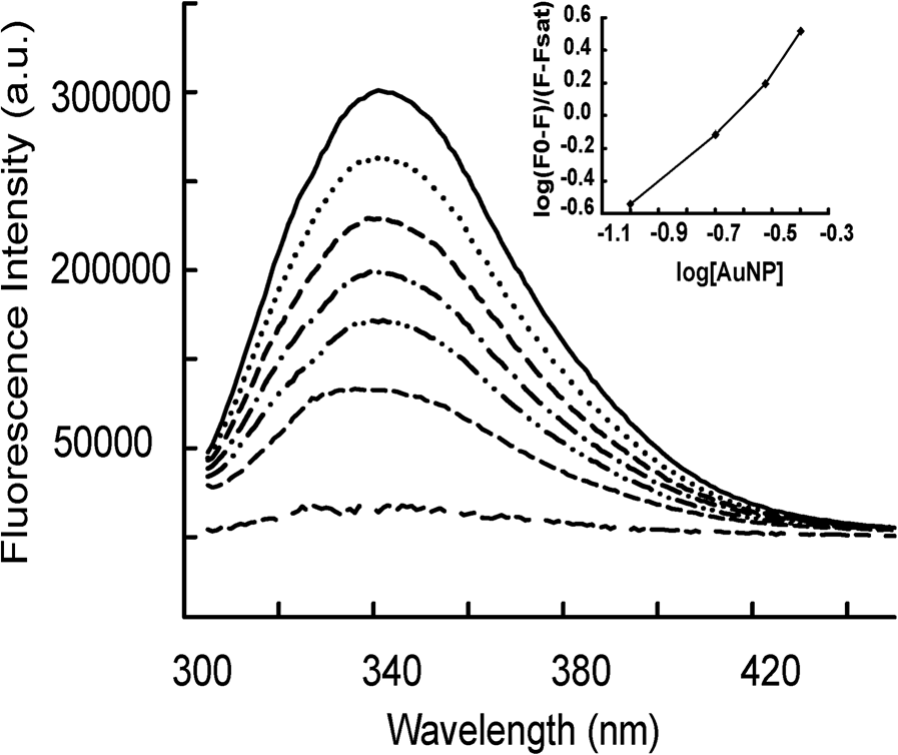
**Effect of AuNP on steady state Tryptophan fluorescence from Gα**_**i1 **_**in inactive state.** In order to monitor the effect of non-covalent interaction of AuNP and Gαi1, intrinsic tryptophan steady state fluorescence was monitored. A final concentration of 400 nM Gα_i1_ in 5 mM Hepes-Na (pH 8.0), 10 mM NaCl, 0.5 mM MgCl_2_, 1 μM GDP was used to monitor the spectrum in all the experiments. The solid line represents 0 nM AuNP; dotted line represents 0.05 nM AuNP; dashed line represents 0.1 nM AuNP; dash-dot-dash line represents 0.2 nM AuNP; dash-dot-dot line represents 0.3 nM AuNP; Short dash-short dash line represents 0.4 nM AuNP; short dash-long dash line represents 0.5 nM AuNP. Inset displays the double-logarimithic plot of the quenching of Gαi1 tryptophan fluorescence related to addition of AuNP. Spectra are representative and experiments were repeated several times. All the spectra were recorded at 25°C.

Tryptophan fluorescence of Gα_i1_ was quenched by AuNP in a dose-dependent (0.1-0.5 nM) manner (Figure 1). No shift was observed in the λ_max,em_ of tryptophan Gα_i1_-AuNP conjugate formation, indicating that the polarity of tryptophan environment, and hence the overall protein structure did not change upon conjugation. The binding constant (K_b_) and the numbers of binding sites (n) between AuNPs and Gα_i1_ were determined using the method described by Tedesco *et al.,*[24] as 7.58 × 10 ^12^ M^-1^ and 1.2 respectively, from the fluorescence spectral titration.

### Rate of activation is differentially affected by the nature of conjugation

Fluoroaluminates activate Gα_i1_-GDP by mimicking the γ-phosphate of GTP in its binding site. Time dependent fluorescence changes from Gα_i1_ upon activation by AlF_4_^-^ binding was monitored for non-covalent and covalent AuNP conjugated Gα_i1_ and activation rates were calculated.

**Figure 2:**
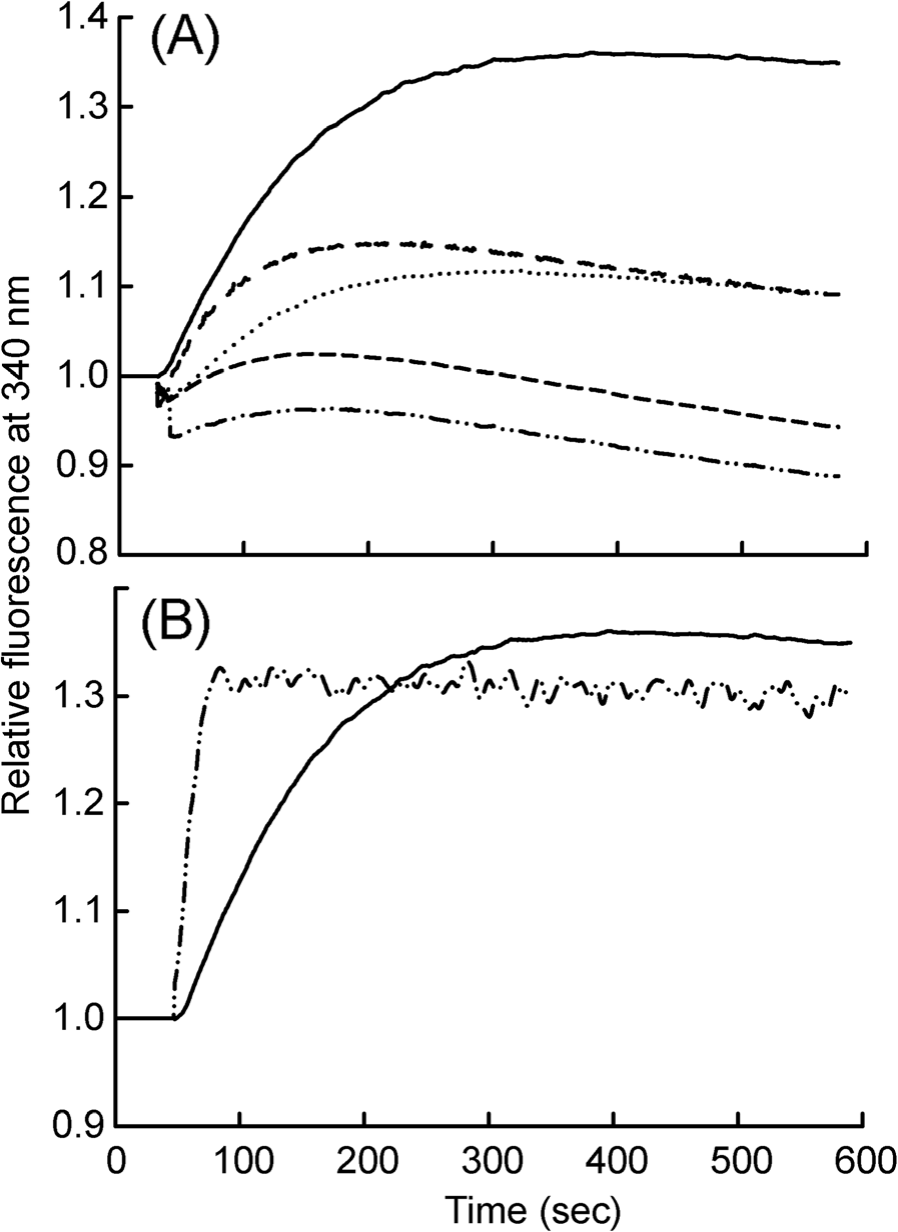
**Time course AlF**^**4- **^**mediated Gα**_**i1 **_**activation of bioconjugated AuNP- Gα**_**i1**_**.** (**A**) Demonstrates effect of non-covalent interaction of AuNP- Gα_i1_ on rate of AlF^4-^ binding to Gα_i1_-GDP. Solid line represents 0 nM AuNP (only protein); Short dash-short dash line represents 0.1 nM AuNP; dotted line represents 0.5 nM AuNP; dashed line represents 0.75 nM AuNP; dash-dot-dot line represents 1 nM AuNP. (**B**) Demonstrates effect of N-terminal covalent conjugation of Gα_i1_ to AuNP *via* EDC reaction on AlF^4-^ binding to AuNP- Gα_i1_-GDP. The solid line represents Gα_i1_ without any conjugation (control); the dash-dot-dot line represents conjugated AuNP- Gα_i1_. For all the time course fluorescence measurement final concentration of 200 nM of Gα_i1_ (conjugated and purified 200 nM AuNP- Gα_i1_ in case of covalent conjugation) was taken in a quartz cuvette containing 5 mM Hepes-Na (pH 8.0), 10 mM NaCl, 0.5 mM MgCl_2_, 1 μM GDP. In case of experiments performed in panel A, appropriate amounts of AuNP-DHLA (in 5 mM Hepes-Na (pH 8.0)) were mixed with protein and incubated for 10 minutes. Tryptophan emission at 340 nm was monitored by exciting the sample at 295 nm with continuous stirring. 2 mM NaF and subsequently followed by 20 μM AlCl_3_ was added to the reaction and relative fluorescence was monitored as a function of time. All the measurements were performed at 25°C. Non-covalently conjugated AuNP- Gα_i1_ displayed deaccelerated rates of basal AlF^4-^ binding to Gα_i1_-GDP. Non covalent conjugates decreased AlF^4-^ binding upto 0.08 fold. On the contrary N-terminal covalent conjugation caused 3.2 fold increase in rate of AlF^4-^ binding. The Plateau fluorescence intensity of covalently conjugated AuNP- Gα_i1_ was comparable to only Gαi1, whereas non-covalent conjugation displayed decrease in plateau fluorescence in a concentration dependent manner.

*(i) Non-covalent conjugation:* Attenuation in the rate of activation accompanied with decrease in values of maximum plateau in fluorescence by AlF_4_^-^ were observed with to non-covalent complex of Au:Gα_i1(GDP)_ . The effect was AuNP concentration dependent, with total loss of activity at 0.4 nM AuNP for 200 nM Gα_i1_ (Figure 2A, Table 1).

**Table 1:** Effect of AuNP on basal rate constants of Gαi1-AlF4- binding

***Non-Covalent AuNP- Gαi1 [AuNP] nM***	***k***_***app ***_***(sec***^***-1***^***)***	**Fold change**
0	2.74	-
0.1	2.34	0.85
0.5	1.12	0.43
0.75	0.004	0.17
1	0.003	0.08
Covalent AuNP- Gαi1	8.8	3.2

*(ii) Covalent conjugation:* Conversely, covalent conjugation at the N-termini of the protein caused enhancement in the rate of AlF^4-^ mediated activation, 3.2 fold in comparison to unconjugated protein (Figure 2B, Table 1).

Both non-covalently and covalently bound AuNP did not perturb the characteristic feature of Gα_i1_ to bind GDP nucleotide and its behaviour to undergo activation-dependent changes induced by transition state mimetic, AlF_4_^-^. Non-covalent and covalent conjugation, modulated kinetics of AlF_4_^-^ induced activation of Gα_i1_ in contrasting manner. The rate of activation by AlF_4_^-^ is much faster in case of covalently conjugated protein and the peak fluorescence of active protein was comparable with respect to unconjugated protein (Figure 2).

### Conjugation does not affect the secondary structure of the protein

Far-UV Circular Dichroism (CD) spectra were recorded to monitor secondary structural features of the protein. Non-covalent or covalent conjugation with AuNP did not cause changes in secondary structure of the protein suggesting the global structure of complex of Au:Gα_i1(GDP)_ to be intact (Additional file 3: Figure S3). These findings clearly indicate that the conjugation of AuNP changes the activity of Gα_i1_ without affecting the conformation of the protein.

### Mode of interaction between Gα_i1_ and AuNP alter the kinetics of basal GTPγS binding

**Figure 3:**
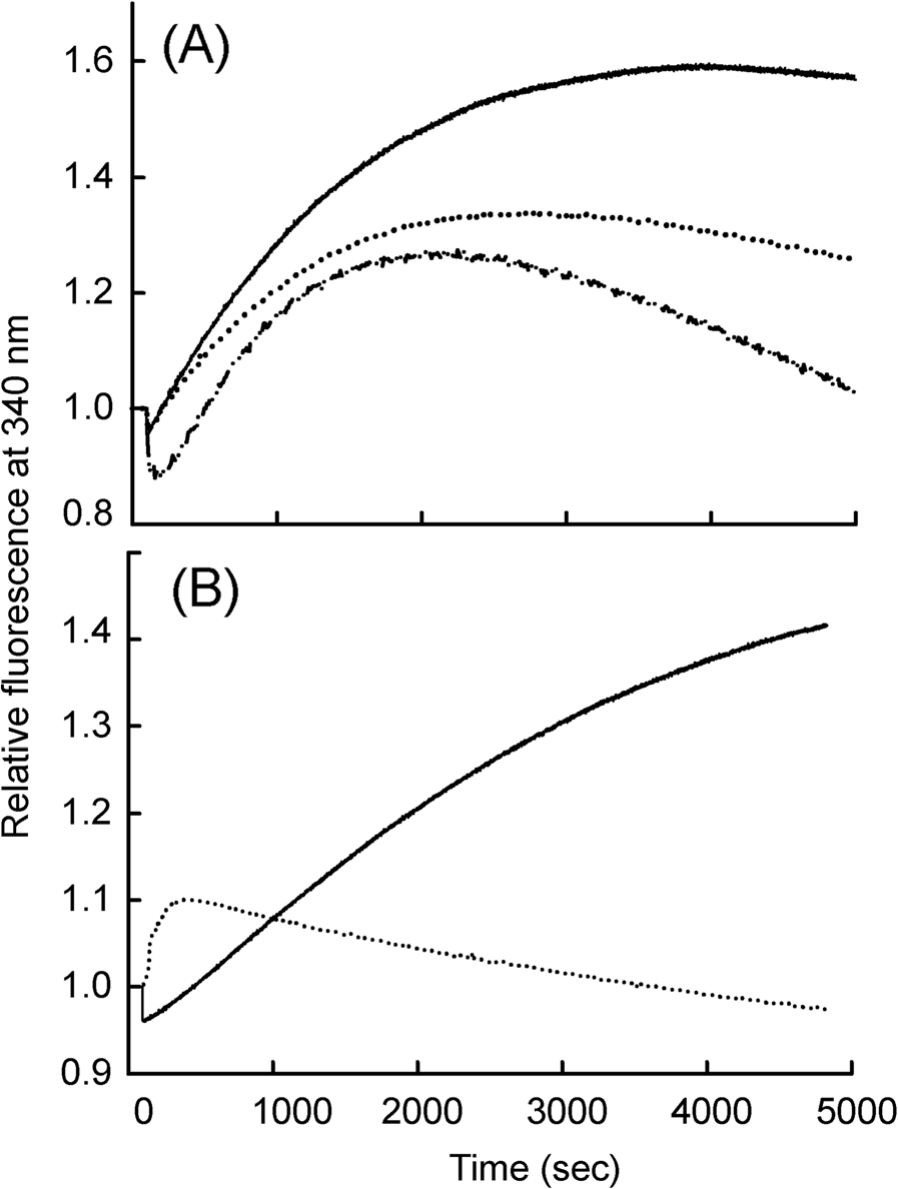
**Effect of AuNP conjugation on Gα**_**i1 **_**basal nucleotide exchange rates.** (**A**) Displays effect of non-covalent interaction between AuNP and Gα_i1_ on basal GTPγS uptake by Gα_i1_-GDP. Solid line represents 0 nM AuNP (only Gα_i1_); Dotted line represents 0.1 nM AuNP; Dash-dot-dot line represents 0.2 nM AuNP. (**B**) Shows effect of N-terminal covalent conjugation of Gα_i1_ to AuNP. Solid line represents 0 nM AuNP (only Gα_i1_); Dotted line represents covalently conjugated AuNP- Gα_i1_. All the experiments were performed with final concentration of 200 nM Gα_i1_ (unconjugated or conjugated) in a quartz cuvette containing 5 mM Hepes-Na (pH 8.0), 10 mM NaCl, 0.5 mM MgCl_2_, 1 μM GDP. Relative tryptophan emission at 340 nm was monitored by exciting the sample at 295 nm with continuous stirring after addition of GTPγS to a final concentration of 3.2 μM. Non-covalently conjugated AuNP- Gα_i1_ displayed deaccelerated rates of basal GTPγS uptake (fold decrease in apparent rates upto 0.8). N-terminal covalent conjugation displayed ~ 5 fold fast basal GTPγS uptake.

Next, we investigated whether functionalization of Gα_i1_ with AuNP affects the basal nucleotide exchange rate, the *in vivo* activity of the protein. Nucleotide exchange (GDP to GTPγS) by Gα_i1_, upon covalent and non-covalent conjugation of AuNP, was monitored by measuring the enhancement in intrinsic Trp fluorescence. Changes in fluorescence were monitored as a function of time after addition of GTPγS (Figure 3, Table 2). Non-covalent conjugation led to a drop in the basal rate of GTPγS uptake, while covalent conjugation caused an increase of ~ 5 fold in the rate of GTPγS uptake. Rate of GTPγS uptake by both types of AuNP:Gα_i1(GDP)_ complexes corroborated GDP/AlF_4_^-^ activation data. Both results provide evidence for dependence of functional behaviour of conjugates on the nature of interaction between AuNP and Gα_i1_, as the conjugates preserved the native conformation confirmed by far UV CD analysis.

**Table 2:** **Effect of AuNP conjugation on Gα**_**i1 **_**basal GTPγS uptake**

**Non-Covalent AuNP- Gα**_**i1 **_**[AuNP] nM**	***k***_***app ***_**(sec**^**-1**^**)**	**Fold change**
0	0.4	-
0.1	0.30	0.75
0.2	0.35	0.87
Covalent AuNP- Gα_i1_	1.9	4.75

### AuNP conjugation modulates Gα_i1_ intrinsic GTPase activity

We used an extrinsic fluorescent probe, N' – Methylanthraniloyl (mant)-GTP (mGTP) in order to quantitatively study effect of AuNP conjugation on binding and release of nucleotide (GTP/GDP) and monitor Gα_i1_ activation. Fluorescence resonance energy transfer (FRET) was monitored as a function of time by exciting the intrinsic tryptophan fluorescence at 295 nm and measuring the mGTP fluorescence at 448 nm. 200 nM Gα_i1_ was titrated with several concentration of mGTP (100 nM to 800 nM). mGTP fluorescence increased upon addition to Gα_i1_ and then decayed at a slower rate, confirming mGTP hydrolysis to mGDP. Further, addition of 10 μM GTPγS decreased the mGDP fluoresecence rapidly. To obtain the corrected mGTP uptake and hydrolysis, fluorescence remaining after GTPγS addition was subtracted.

**Figure 4:**
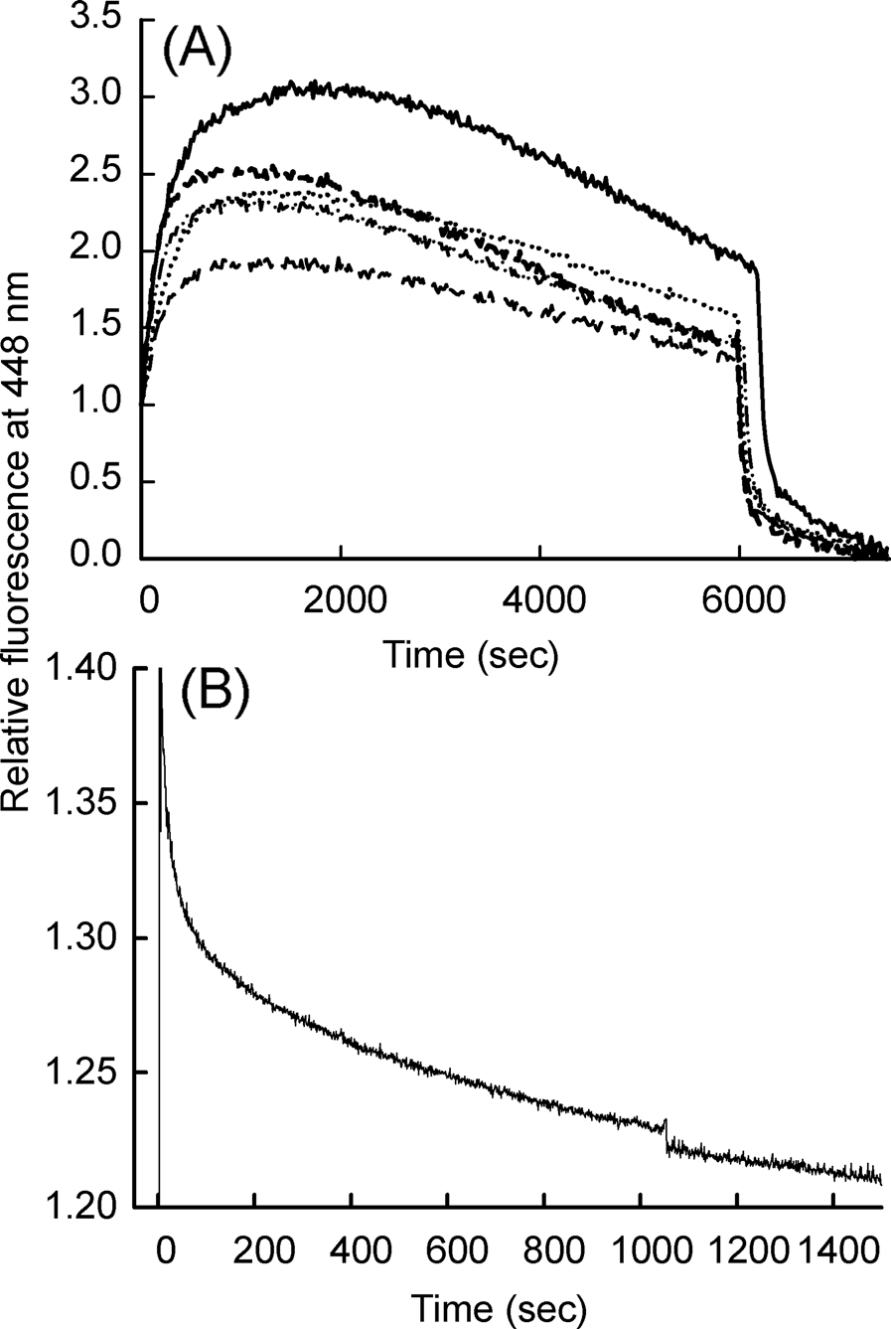
**FRET based time course measurement to study effect of AuNP conjugation on Gα**_**i1 **_**basal nucleotide uptake and hydrolysis.** Fluorescently-labeled nucleotide (mant-GTP) was used to do FRET studies for monitoring rates of nucleotide exchange. (**A**) Displays effect of non-covalently conjugated AuNP- Gα_i1_ on FRET based measurements. The solid line represents 0 nM AuNP; Short-short-short line represents 0.1 nM AuNP; Dash-dot-dash line represents 0.2 nM AuNP; Dotted line represents 0.3 nM AuNP; Long-short line represents 0.4 nM AuNP. (**B**) Displays effect of N-terminal covalent conjugation on FRET based measurements. The solid line represents covalently conjugated AuNP-Gα_i1_. In all the experiments, a final concentration of 200 nM Gα_i1_ was taken in a quartz cuvette containing 5 mM Hepes-Na (pH 8.0), 10 mM NaCl, 0.5 mM MgCl_2_, and 1 μM GDP. Time course FRET was monitored by exciting the protein intrinsic tryptophan (λ_ex_ 295 nm, λ_em_ 340 nm) and monitoring fluorescence from MANT (λ_ex_ 355 nm, λ_em_ 448 nm). 700 nM MANT-GTP was added and relative increase in fluorescence was monitored as a function of time. At 6000 seconds, 10 μM GTPγS was added to the reaction mixture. The fluorescence remaining after addition of GTPγS was subtracted from the data. Non-covalent interaction resulted in retardation in basal Gα_i1_ mGTP uptake in a AuNP concentration dependent manner.

**Figure 5:**
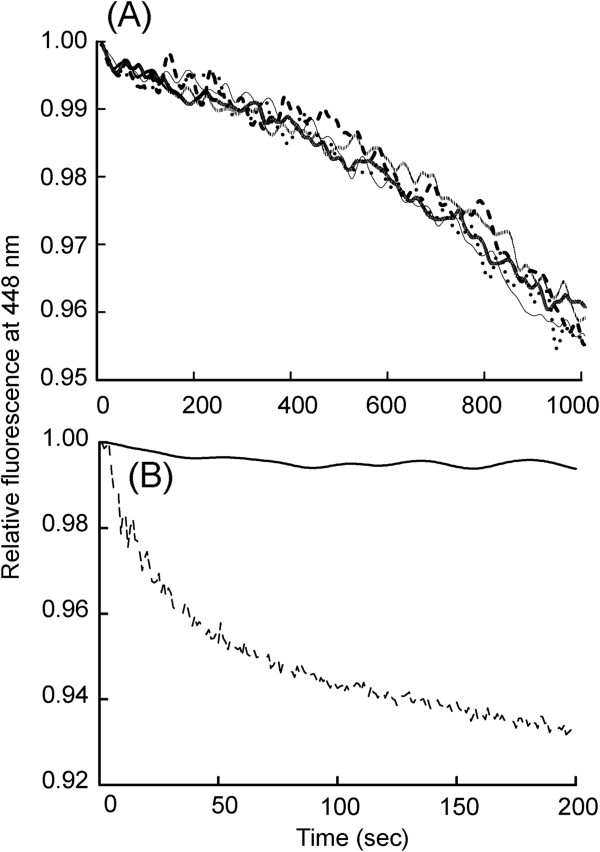
**FRET based time course measurement to study effect of AuNP conjugation on Gα**_**i1 **_**intrinsic GTPase activity by monitoring GTP hydrolysis.** (**A**) Illustrates effect of non-covalently conjugated AuNP-Gαi1 on intrinsic GTPase activity. The thin solid line represents 0 nM AuNP; dotted line represents 0.1 nM AuNP; thick dark line represents 0.2 nM AuNP; dashed line represents 0.3 nM AuNP; line with grey bars represents 0.4 nM AuNP. (**B**) Display effect of N-terminal covalent conjugation on intrinsic GTPase activity. The solid line represents only Gαi1; dashed line represents covalently conjugated AuNP-Gα_i1_. In all the experiments, a final concentration of 200 nM Gα_i1_ was taken in a quartz cuvette containing 5 mM Hepes-Na (pH 8.0), 10 mM NaCl, 0.5 mM MgCl_2_, and 1 μM GDP. Non-covalent conjugation retarded the mGTP hydolysis rate. The mGTP hydrolysis rates were reduced upto 0.8 fold. N-terminal covalent conjugation drastically increases the mGTP hydrolysis rate by 12.16 fold.

Non-covalent conjugation of Gα_i1_ with AuNP retarded the basal exchange rate of mGTP to protein bound GDP, up to 0.4 fold, in a concentration dependent manner of AuNP (Figure 4A, Table 3) Altered Gα_i1_-NP basal nucleotide uptake convincingly demonstrates AuNP influence of the mode of binding. Further, to investigate the effect of AuNP conjugation on Gα_i1_ GTPase function mGTP hydrolysis kinetics was monitored. Significant decrease in mGTP hydrolysis rate (Figure 5A) was observed (Table 4), suggesting the non-covalent binding with AuNP has an inhibiting effect on intrinsic GTPase property of Gα_i1_. Covalent conjugation of Gα_i1_ with AuNP resulted in 12 fold increase in GTPase activity (Figure 4B). This indicates that covalent conjugation of AuNP to Gα_i1_ has an accelerating effect on intrinsic GTPase function of Gα_i1_.

**Table 3:** **Effect of AuNP conjugation on Gα**_**i1 **_**basal mGTP uptake**

**Non-Covalent AuNP- Gα**_**i1 **_**[AuNP] nM**	***k***_***app ***_**(sec**^**-1**^**)**	**Fold change**
0	7.6	-
0.1	7.5	0.98
0.2	6.0	0.80
0.3	4.0	0.53
0.4	3.3	0.44

**Table 4:** **Effect of AuNP conjugation on Gα**_**i1 **_**GTPase activity**

**Non-Covalent AuNP- Gα**_**i1 **_**[AuNP] nM**	***k***_***app ***_**(sec**^**-1**^**)**	**Fold change**
	0.46	-
0.1	0. 43	0.934
0.2	0.40	0.869
0.3	0.40	0.869
0.4	0.37	0.804
Covalent AuNP- Gα_i1_	4.5	12.16

## Discussion

In the present study, we address the dependence on the chemistry of conjugation towards alteration in the kinetics of activation of Gα_i1_. There have been contradicting observations regarding the benefit of using a nanoparticle in medicine and biochemistry [25-27]. In our view, understanding the physico-chemical basis of how an engineered nanoparticle modulates biological processes requires the study of nanoparticle-biomolecule binding and its effects on biomolecule functionality. Recent studies have emphasized that properties like size, shape, surface modification, and charge of nanoparticles can profoundly affect the interaction between NPs and biomolecules.

Guanine nucleotide binding proteins (G-protein) play a vital role in the physiology of a cell. Structure-function relationship of both, the monomeric and the heterotrimeric G proteins are well understood, their crystal structures helping elucidate their mode of action and the biochemical function. Heterotrimeric G-proteins are activated by agonist-stimulated G Protein-Coupled Receptors (GPCRs) that catalyze the exchange of GTP for GDP on G protein α-subunits and relay extracellular signal to intracellular signalling pathways [28]. We chose to use G proteins as model proteins to better understand the effect of AuNP binding to proteins and the biological effect they elucidate.

Here, we exploit two modes of conjugation between Gα_i1_ and AuNP, covalent and non-covalent. Effect of AuNP conjugation to Gα_i1_ was examined by monitoring steady-state Trp-fluorescence from the protein. AuNP interacts with Gα_i1_ with a binding constant (K_b_) of 7.58 × 10 ^12^ M^-1^. Strong tryptophan fluorescence quenching of Gα_i1_ was observed with increasing concentration of AuNP. Fluorescence quenching could be explained by efficient energy transfer between AuNP and Gα_i1_ tryptophan residues. No shift in emission wavelength was noticed, suggesting no change in polarity around tryptophan residues on addition of AuNPs.

Cysteine modifications did not alter the AuNP conjugation and, vice versa, conjugation did not lead to the unavailability of the thiol groups of cysteine for the modifying reagents, therefore leading us to conclude that the 10 cysteine residues (of Gα_i1_) do not interact with AuNP via thiol-Au linkage chemistry. Our finding is in agreement with a previous study which demonstrates that cysteine residue at the end of a C-terminus of protein was much more reactive toward a gold cluster than a cysteine residue introduced in middle region of protein [29]. This study concludes that non-specific AuNP interaction is not protein-sulphydryl mediated even though cysteine residues are present on Gα_i1_ surface.

A number of studies have shown that nanoparticle protein conjugates undergo conformational changes and result in unfolding of protein [30-32]. For biochemical applications of NP-protein conjugates, it is crucial that labelling does not modify the protein structure. Interestingly, both types of conjugates of Gα_i1_ retain their secondary structure as evident from far UV-CD spectra profile for Gα_i1_ and the conjugates (Additional file 3: Figure S3).

We next investigated whether the bioconjugated Gα_i1_ was functional and active. We report here functional activity of both the covalent and non-covalent AuNP conjugated Gα_i1_. Time dependent fluorescence measurement using intrinsic tryptophan and extrinsic MANT moiety fluorescence with hydrolyzable and non-hydrolyzable nucleotides were assayed. Detailed kinetics based functional studies for both non-covalent and covalent AuNP-Gα_i1_ conjugates have provided important insights: *(i)* reduced rate of activation by AlF^4-^, GTPγS and mant-GTP were observed as a consequence of non-covalent interaction of AuNP. *(ii)* N-terminal covalent probing led to enhanced rate of nucleotide uptake “activity” of Gα_i1_.

**Figure 6:**
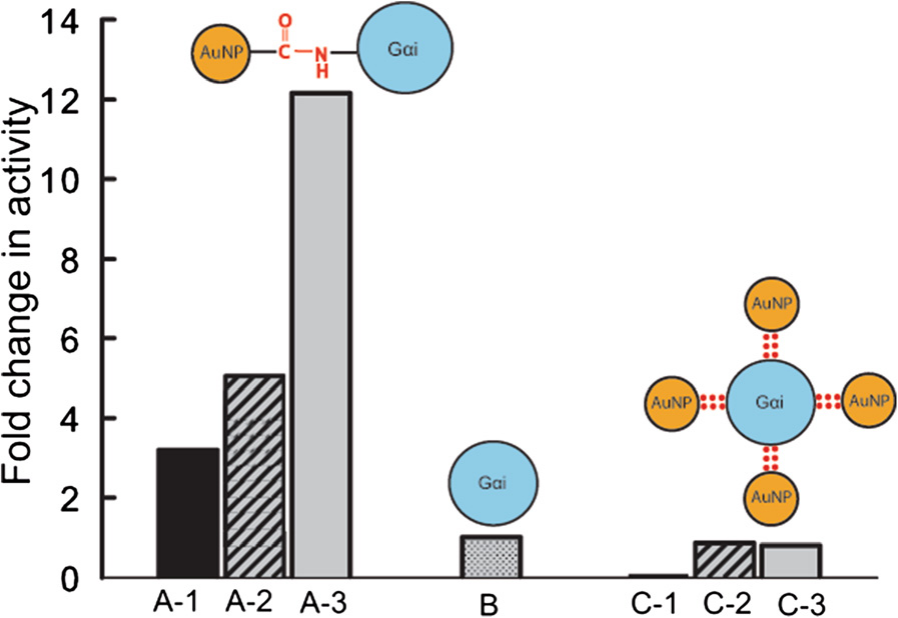
**Schematic illustration of consequence of AuNP conjugation on Gαi1 function.** Group (**A**) displays fold change by covalent conjugate for binding of AlF^4-^ (A-1), uptake of GTPγS (A-2), GTP hydrolysis (A-3) (**B**) corresponds to unconjugated Gα_i1_. Group (C) displays fold change by non-covalent conjugate for binding of AlF^4-^ (C-1), uptake of GTPγS (C-2), GTP hydrolysis (C-3).

To further probe the influence of AuNP conjugation on intrinsic Gα_i1_ GTPase function, kinetics of bound GTP hydrolysis was examined. As demonstrated in Figure 5A, non-covalent conjugation does not affect the GTPase function while covalent conjugation dramatically accelerated it. From our studies, it may be hypothesized that the functional property of conjugated protein are governed by the contribution of type of molecular interaction, between the nanomaterial and biomolecule (Figure 6). This has strong implication that nanoparticles can impair the cell function when it enters into biological fluid depending on the extent and format of presentation of the signalling protein to the nanoparticles. Thus, these finding presented here need to be considered carefully before using engineered nanoparticles for medical application.

## Conclusion

In summary, we here report two different bioconjugation strategies, non-covalent and covalent attachment, of Gα_i1_ to 6 nm DHLA capped AuNP. No effect of change in protein conformation was observed despite the presence of negatively charged capping ligand, DHLA. Non-covalent bioconjugation caused decrease in “activity” of Gα_i1_ in terms of decelerated rate of nucleotide exchange and inhibited GTPase activity. N-terminal covalent probing of AuNP modulate the *active state* of Gα_i1_ state, as displayed by enhanced rate of nucleotide exchange and stimulated GTPase function. These results (extraordinary increase in the Gα_i1_ GTPase property) have ramification in understanding the probable molecular basis of gold to cure many diseases when used either in powder form (in ayurvedic treatment) or as colloidal gold in modern medicine (e.g., in arthritis).

## Methods

### Materials

All the chemicals used were purchased from Sigma-Aldrich, USA.

### Synthesis of Gold nanoparticles (AuNPs)

The synthesis protocol of 6 nm gold nanoparticles is adopted from Nikhil and Xiaogang (2003) for a single-phase reaction (Additional file 4: S4) [33].

### Size and optical characterization of AuNP

Transmission electron microscopy was used to visualise the shape and to determine size distribution of AuNPs (Additional file 5: Figure S5). TEM images were obtained using JEOL 3010, operating at 300 kV accelerating voltage. The average size distribution was determined by using the image analysis software, Image J. The UV–vis absorption spectra were recorded on a Jasco V-660 UV–vis spectrometer at room temperature with 1 cm path length cuvette. Spectra were obtained with a band width of 1.0 nm and a scan rate of 40 nm/minutes (Additional file 6: Figure S6). Toluene was used as reference.

### Expression and purification of Gα_i1_ protein

The DNA fragment containing the WT rat Gα_i1_ subunit, cloned into the pET28b expression vector was used to transform BL21 (DE3) cells to express an N-terminal hexa-His-tag-WTGαi1 protein in the presence of kanamycin (100 μg/mL) and purified (Additional file 7: S7). The eluted protein fractions with the maximum protein content were estimated by Lowry’s method [34] and purity checked by SDS-PAGE. The average yield for WT Gα_i1_ was 10 mg/L of culture.

### Bioconjugation

(i) *Non covalent conjugation*: 100 μL, 0.2 μM purified AuNP-DHLA (in 5 mM Hepes-Na, pH 8.0) was incubated with 100 μL, 200 μM Gα_i1_ at room temperature for 20 minutes. Unconjugated protein and free AuNP were removed by centrifugation (12000 rpm, 20 minutes 4°C) (Additional file 1: Figure S1A).

(ii) *Covalent bioconjugation*: 1-Ethyl-3-[3-dimethylaminopropyl] carbodiimide (EDC) was used as a cross linking agent between carboxyl groups of AuNP and primany amines of Gα_i1_. For linkage, 100 μL 3 mM EDC (prepared in double distilled water) was added to 100 μL 0.2 μM AuNP and mixed. 100 μL, 200 μM Gα_i1_ was added to the mixture containing AuNP and EDC and incubated for 15 minutes at room temperature. Unbound protein and AuNP were removed by centrifugation (15 min, 14000 rpm). Gel electrophoresis was used to confirm conjugation (Additional file 1: Figure S1A).

### Fluorescence spectroscopy

#### Steady state fluoresecence

Steady state intrinsic tryptophan fluorescence of Gα_i1_ in inactive form was recorded on HORIBA Jobin Yvon fluorolog spectrometer with excitation light of 295 nm (excitatoin and emission slit width of 5 nm) at 25°C. In a 3 mL quartz cuvette, 400 nM Gα_i1_ [in 5 mM Hepes-Na (pH 8.0), 10 mM NaCl, 0.5 mM MgCl_2_ and 1 μM GDP] was taken and titrated with AuNPs. In all cases blank spectra (buffer containing only AuNP) were subtracted from the protein spectra.

#### Fluorescence-based kinetic assays

Time-based fluorescence activity measurements were performed on a Jasco FP-6500 Spectrofluorometer at 25°C. In a 3 mL cuvette, 400 nM Gα_i1_ [in 5 mM Hepes (pH 8.0), 10 mM NaCl, 0.5 mM MgCl_2_ and 1 μM GDP] was taken. 16 μM GTPγS was added to the protein and the relative increase in intrinsic fluorescence (λex = 295 nm, λem = 340 nm) was measured as a function of time. Similar measurments were performed for AuNP conjugated Gα_i1_. GTPγS exchange rates were determined as described elsewhere [35]. FRET was monitored by exciting the intrinsic Trp fluorescence at 295 nm and measuring the mant-GTP fluorescence at 448 nm. In all cases, blank spectra containing buffer alone were subtracted from the final spectra.

#### Circular dichroism (CD) spectroscopy

CD measurements were made on a JASCO model J-715 spectropolarimeter. Far-UV-CD spectra were recorded in 1 cm path length cuvette from 200 to 260 nm; each spectrum was the average of 5 scans. Spectra were recorded with the final protein concentration of 100 μg/mL. Appropriate buffer spectra were recorded and subtracted from the protein spectra.

#### Data analysis

The apparent rate constants (*kapp*) reported (Table 1, 2, 3) is the mean of several independent experiments and represent *kapp* x 10^-3^ sec^-1^. Initial 1000 (for non-covalent conjugation) and 200 (covalent conjugation) data points were used to calculate the apparent rate constants reported. In Table 4, the apparent rate constants (*kapp*) reported is the mean of several independent experiments and represents *kapp* x-10^-4^ sec^-1^. The -fold change in the rate of the AuNP- Gα_i1_ to that of Gα_i1_ is calculated as *kapp*(AuNP- Gα_i1_)/*kapp*(Gα_i1_).

## Competing interests

The authors declare that they have no competing interests.

## Authors’ contribution

SPN and GKA designed research; SV performed research; SV, SPN and GKA analyzed data; and SV, SPN and GKA wrote the paper. All authors read and approved the final manuscript.

## Supplementary Material

Additional file 1: Figure S1 (A) Bioconjugation of AuNP-DHLA to Gαi1 via EDC resulting in a covalent linkage. Agarose gel (2%) of gold nanoparticles with and without proteins attached to them. Lane1: AuNP (control), Lane (2): AuNP + EDC (negative control), Lane (3), (4), (5): covalently conjugated AuNP- Gαi1 with 100, 200, 300 μM Gαi1 respectively. Retardation in electrophoretic mobility in lanes (3), (4) and (5) is attributed to formation of bioconjugates. AuNP in presence of EDC (Lane 2) also showed little retardation in mobility even when no protein was present. This could be explained by formation of O-acylisourea intermediates formed between AuNP and EDC. (B) Bioconjugation of AuNP-DHLA to Gαi1 via non-covalent interaction. Lane (1): AuNP (control), Lane (2): AuNP- Gαi1 (electrostatic interaction), Lane (3): AuNP- Gαi1 where Gαi1 Cysteines were modified by Iodoacetamide before conjugation. Retardation in electromobility in lanes (2) and (3) confirms bioconjugation. No difference in mobility in lanes (2) and (3) rules out thiol-AuNP interaction. Click here for file

Additional file 2: S2 Cysteine modification: Iodoacetamide was used to derivatize cysteines in Gαi1. 50 μL, 100 μM Gαi1 was incubated with 10 μL, 100 mM Iodoacetamide (in 5 mM Hepes-Na, pH 8.0) for 15 minutes at 25°C. Complete cysteine alkylation was monitored by 5,5'-dithiobis-(2-nitrobenzoic acid) [DTNB] assay. Standard plot was obtained using Gαi1 from 1-10 μM. To check for free cysteine groups in AuNP conjugated Gαi1 fluorescence adducts were formed with N-(3-pyrene) maleimide and emission spectra was recorded with Excitation light of 345 nm. Click here for file

Additional file 3: Figure S3 Far UV Circular Dichroism (CD) spectra of AuNP- Gαi1 conjugates. (A) Displays far-UV CD spectra of non-covalently conjugated AuNP-Gαi1. The solid line represents 400 nM Gαi1 only (without AuNP); Dotted line represents 400 nM Gαi1 with 0.6 nM AuNP; dash-dash-dash line represents 400 nM Gαi1 with 1 nM AuNP. (B) Displays far-UV CD spectra of N-terminal covalently conjugated AuNP- Gαi1. Click here for file

Additional file 4: S4 Synthesis protocol of AuNP. In brief, 462.62 mg Didodecyldimethylammonium bromide (DDAB) was dissolved in 10 mL toluene and 86.135 mg decanoic acid was dissolved 5 mL toluene to give stock solution of 100 mM. Gold precursor solution (25 mM) was prepared by dissolving 6.8 mg of gold (III) chloride (AuCl3) in 0.8 mL 100 mM DDAB solution. In a typical synthesis, 1 ml of freshly prepared Tetrabutylammonium borohydride (TBAB) solution (25.73 mg in 1 mL of DDAB solution) was mixed with 0.625 ml decanoic acid stock solution under vigorous stirring and 0.8 ml gold precursor solution was injected leading instantaneously to a dark-red solution of Gold nanoparticles (AuNPs) capped with DDAB. After two hours the solution was centrifuged (2500 rpm, 30 min) to remove free surfactants, reducing agents and smaller nanoparticles. The precipitate of AuNPs was then re-dissolved in 2.5 ml DDAB stock solution. Ligand exchange: To a 2.5 mL solution of AuNP-DDAB freshly reduced 0.104 mg lipoic acid (LA) was added and stirred until no bubbles generated. The brown precipitate of AuNP-DHLA was purified by washing with toluene and chloroform and all solvents were evaporated. Addition of 5 mL of 0.1 M NaOH caused deprotonation of the COOH groups of the dried AuNP-DHLA and thus rendering the AuNP soluble in the water phase. The gold nanoparticles were purified by passing through the membrane of a 30 KDa molecular weight cut-off (MWCO) centrifuge filter (Millipore) and the particles were concentrated, followed by buffer exchange. Click here for file

Additional file 5: Figure S5 High resolution Transmission electron microscopic (HRTEM) images of AuNP-DHLA. Sample was diluted and directly added on carbon-coated copper TEM grids and the solvent evaporated to form a dry particle film. Images confirm very narrow size distribution. Scale bar corresponds to 50 nm, 20 nm, 5 nm for panel A, B and C respectively. 50 particles were randomly selected and size distribution was measured using Image-J software, resulting in 5.92 nm ± 0.5219 in diameter. Click here for file

Additional file 6: Figure S6 Surface plasmon resonance of AuNP. UV-vis absorption spectra of the as-prepared gold nanoparticles (AuNP-DDAB, dotted lines) and after ligand exchange (AuNP-DHLA, solid line). Au samples with DDAB capping were dissolved in toluene, sample with DHLA capping in aqueous solution. AuNP capped with DDAB showed strong plasmon resonance in the range of 520-530 nm. Plasmon resonance was preserved after ligand exchange with DHLA. This confirms stability of AuNP in aqueous medium. Click here for file

Additional file 7: S7 Purification of Gαi1 protein. Cells were grown at 37°C to A600 nm of ~ 0.7 and then induced with 100 μM isopropyl-β-D-thiogalactoside (IPTG). The culture was then grown for 16 hours at 23°C. Cells were harvested by centrifugation, and the resulting pellets were resuspended in a buffer containing 20 mM Tris–HCl (pH 8.0), 300 mM NaCl, 2 mM MgCl2, 10 μM GDP. For purification, the cells were sonicated using an ultrasonicator (Vibracell Sonics and Materials, Inc. Newtown, CT, USA). The lysate was centrifuged at 4°C (45 min at 12,000 rpm). The resulting supernatant was loaded onto a nickel nitrilotriacetic acid (Ni–NTA) Superflow resin column (Qiagen, Hilden, Germany) that was equilibrated with 20 mM Tris–HCl (pH 8.0), 300 mM NaCl, 2 mM MgCl2, 10 μM GDP buffer. The protein loaded resin was washed with 10 column volumes with wash buffer 1 [20 mM Tris–HCl (pH 8.0), 300 mM NaCl, 2 mM MgCl2, 10 mM imidazole] and then with 5 column volume with wash buffer 2 [20 mM Tris–HCl (pH 8.0), 300 mM NaCl, 2 mM MgCl2, 30 mM imidazole]. The bound protein was eluted with 2 column volumes of elution buffer [20 mM Tris–HCl (pH 8.0), 300 mM NaCl, 2 mM MgCl2, 10 μM GDP 300 mM imidazole]., were pooled and concentrated to a volume of 1 mL and loaded onto a Superdex 200 26/60 column (GE Healthcare) that was equilibrated in buffer [5 mM Hepes-Na (pH 8.0), 10 mM NaCl, 0.5 mM MgCl2, 1 μM GDP]. After elution, the protein-containing fractions were pooled, concentrated and stored at −80°C. Click here for file
