# Supplementary material for: Chemistry of conjugation to gold nanoparticles affects G-protein activity differently
Source: J Nanobiotechnology. 2013 Mar 19;11:7. doi: 10.1186/1477-3155-11-7 (PMC3614441; doi:10.1186/1477-3155-11-7)
Supplement: Additional file 1: Figure S1 — (A) Bioconjugation of AuNP-DHLA to Gαi1 via EDC resulting in a covalent linkage. Agarose gel (2%) of gold nanoparticles with and without proteins attached to them. Lane1: AuNP (control), Lane (2): AuNP + EDC (negative control), Lane (3), (4), (5): covalently conjugated AuNP- Gαi1 with 100, 200, 300 μM Gαi1 respectively. Retardation in electrophoretic mobility in lanes (3), (4) and (5) is attributed to formation of bioconjugates. AuNP in presence of EDC (Lane 2) also showed little retardation in mobility even when no protein was present. This could be explained by formation of O-acylisourea intermediates formed between AuNP and EDC. (B) Bioconjugation of AuNP-DHLA to Gαi1 via non-covalent interaction. Lane (1): AuNP (control), Lane (2): AuNP- Gαi1 (electrostatic interaction), Lane (3): AuNP- Gαi1 where Gαi1 Cysteines were modified by Iodoacetamide before conjugation. Retardation in electromobility in lanes (2) and (3) confirms bioconjugation. No difference in mobility in lanes (2) and (3) rules out thiol-AuNP interaction. [file 1477-3155-11-7-S1.doc]

**
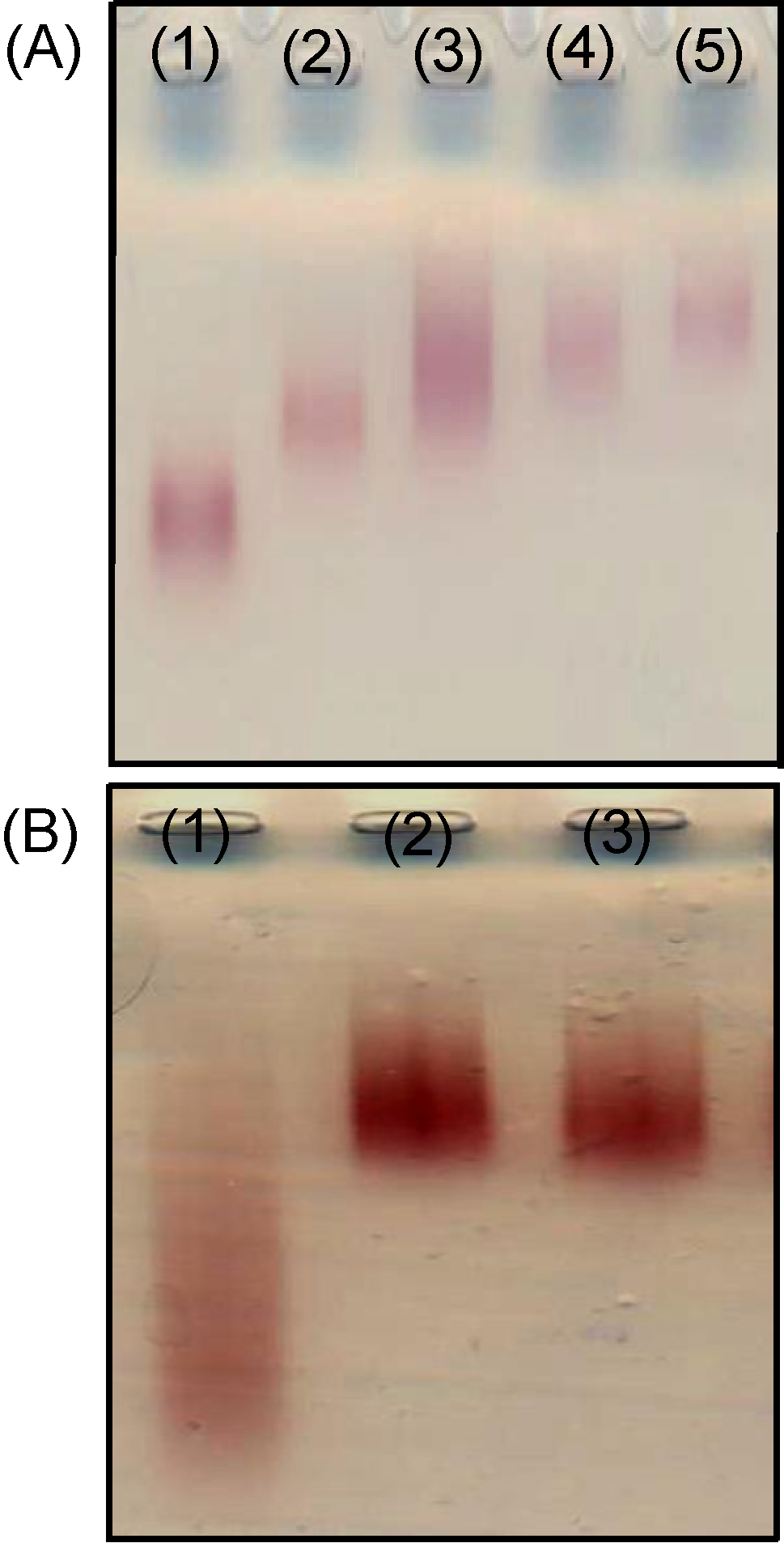
**

**Figure S1:** (A) **Bioconjugation of AuNP-DHLA to Gαi1 *via* EDC resulting in a covalent linkage.** Agarose gel (2%) of gold nanoparticles with and without proteins attached to them. Lane1: AuNP (control), Lane (2): AuNP+EDC (negative control), Lane (3), (4), (5): covalently conjugated AuNP-Gαi1 with 100, 200, 300 µM Gαi1 respectively. Retardation in electrophoretic mobility in lanes (3), (4) and (5) is attributed to formation of bioconjugates. AuNP in presence of EDC (Lane 2) also showed little retardation in mobility even when no protein was present. This could be explained by formation of O-acylisourea intermediates formed between AuNP and EDC.

(B) Bioconjugation of AuNP-DHLA to Gαi1 *via* non-covalent interaction. Lane (1): AuNP (control), Lane (2): AuNP-Gαi1 (electrostatic interaction), Lane (3): AuNP- Gαi1 where Gαi1 Cysteines were modified by Iodoacetamide before conjugation. Retardation in electromobility in lanes (2) and (3) confirms bioconjugation. No difference in mobility in lanes (2) and (3) rules out thiol-AuNP interaction.
