# Supplementary material for: Chemistry of conjugation to gold nanoparticles affects G-protein activity differently
Source: J Nanobiotechnology. 2013 Mar 19;11:7. doi: 10.1186/1477-3155-11-7 (PMC3614441; doi:10.1186/1477-3155-11-7)
Supplement: Additional file 2: S2 — Cysteine modification: Iodoacetamide was used to derivatize cysteines in Gαi1. 50 μL, 100 μM Gαi1 was incubated with 10 μL, 100 mM Iodoacetamide (in 5 mM Hepes-Na, pH 8.0) for 15 minutes at 25°C. Complete cysteine alkylation was monitored by 5,5'-dithiobis-(2-nitrobenzoic acid) [DTNB] assay. Standard plot was obtained using Gαi1 from 1-10 μM. To check for free cysteine groups in AuNP conjugated Gαi1 fluorescence adducts were formed with N-(3-pyrene) maleimide and emission spectra was recorded with Excitation light of 345 nm. [file 1477-3155-11-7-S2.doc]

**S2: Cysteine modification:** Iodoacetamide was used to derivatize cysteines in Gα_i1_. 50 µL, 100 µM Gαi1 was incubated with 10 µL, 100 mM Iodoacetamide (in 5 mM Hepes-Na, pH 8.0) for 15 minutes at 25° C. Complete cysteine alkylation was monitored by 5,5'-dithiobis-(2-nitrobenzoic acid) [DTNB] assay. Standard plot was obtained using Gα_i1_ from 1-10 µM. To check for free cysteine groups in AuNP conjugated Gα_i1_ fluorescence adducts were formed with N-(3-pyrene) maleimide and emission spectra was recorded with Excitation light of 345 nm.
