# Supplementary material for: Chemistry of conjugation to gold nanoparticles affects G-protein activity differently
Source: J Nanobiotechnology. 2013 Mar 19;11:7. doi: 10.1186/1477-3155-11-7 (PMC3614441; doi:10.1186/1477-3155-11-7)
Supplement: Additional file 3: Figure S3 — Far UV Circular Dichroism (CD) spectra of AuNP- Gαi1 conjugates. (A) Displays far-UV CD spectra of non-covalently conjugated AuNP-Gαi1. The solid line represents 400 nM Gαi1 only (without AuNP); Dotted line represents 400 nM Gαi1 with 0.6 nM AuNP; dash-dash-dash line represents 400 nM Gαi1 with 1 nM AuNP. (B) Displays far-UV CD spectra of N-terminal covalently conjugated AuNP- Gαi1. [file 1477-3155-11-7-S3.doc]

**
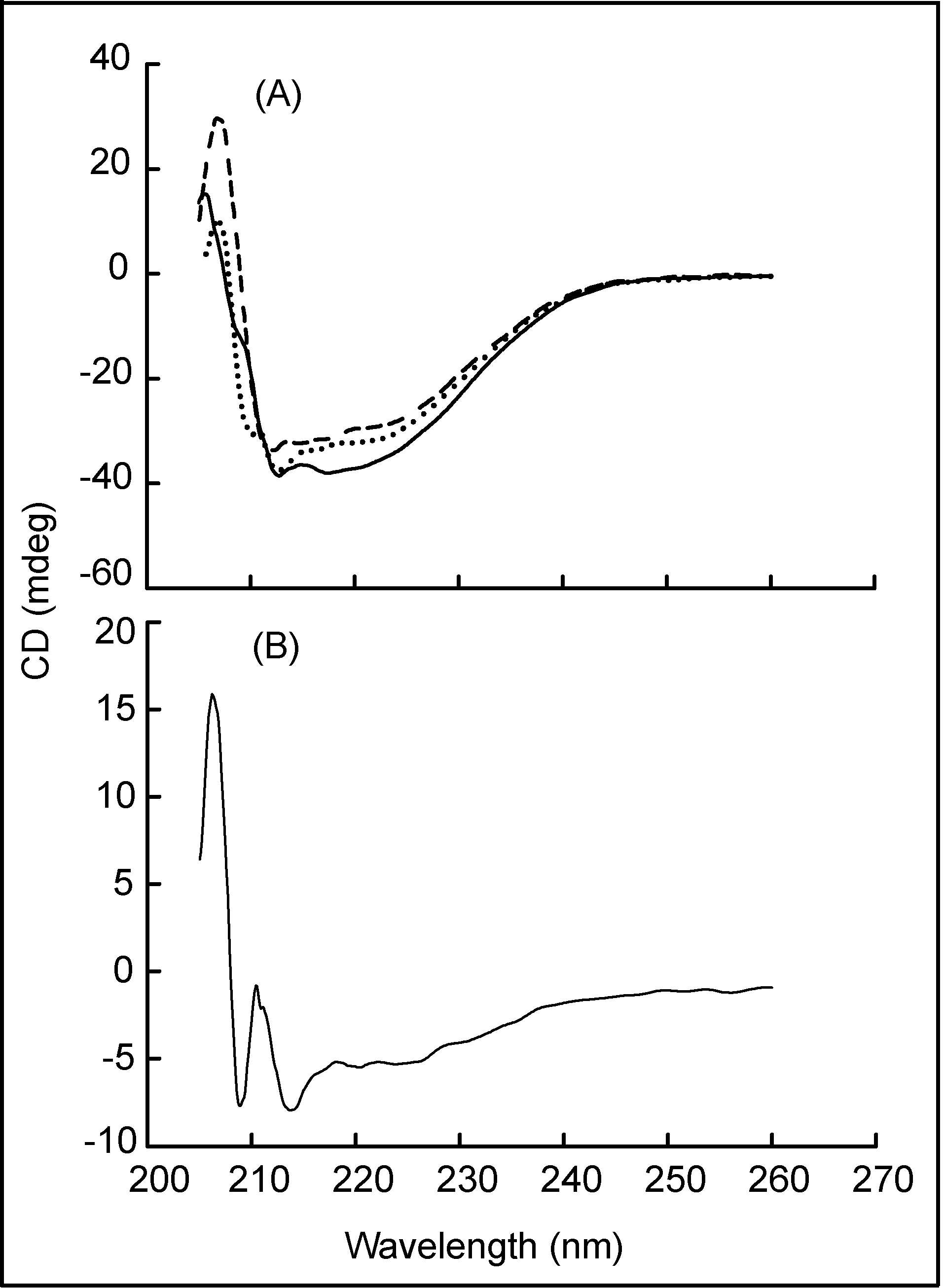
**

**Figure S3: Far UV Circular Dichroism (CD) spectra of AuNP- Gα_i1_ conjugates.** (A) Displays far-UV CD spectra of non-covalently conjugated AuNP-Gα_i1_. The solid line represents 400 nM Gα_i1_ only (without AuNP); Dotted line represents 400 nM Gα_i1_ with 0.6 nM AuNP; dash-dash-dash line represents 400 nM Gα_i1_ with 1 nM AuNP. (B) Displays far-UV CD spectra of N-terminal covalently conjugated AuNP-Gα_i1_.
