# Supplementary material for: Chemistry of conjugation to gold nanoparticles affects G-protein activity differently
Source: J Nanobiotechnology. 2013 Mar 19;11:7. doi: 10.1186/1477-3155-11-7 (PMC3614441; doi:10.1186/1477-3155-11-7)
Supplement: Additional file 4: S4 — Synthesis protocol of AuNP. In brief, 462.62 mg Didodecyldimethylammonium bromide (DDAB) was dissolved in 10 mL toluene and 86.135 mg decanoic acid was dissolved 5 mL toluene to give stock solution of 100 mM. Gold precursor solution (25 mM) was prepared by dissolving 6.8 mg of gold (III) chloride (AuCl3) in 0.8 mL 100 mM DDAB solution. In a typical synthesis, 1 ml of freshly prepared Tetrabutylammonium borohydride (TBAB) solution (25.73 mg in 1 mL of DDAB solution) was mixed with 0.625 ml decanoic acid stock solution under vigorous stirring and 0.8 ml gold precursor solution was injected leading instantaneously to a dark-red solution of Gold nanoparticles (AuNPs) capped with DDAB. After two hours the solution was centrifuged (2500 rpm, 30 min) to remove free surfactants, reducing agents and smaller nanoparticles. The precipitate of AuNPs was then re-dissolved in 2.5 ml DDAB stock solution. Ligand exchange: To a 2.5 mL solution of AuNP-DDAB freshly reduced 0.104 mg lipoic acid (LA) was added and stirred until no bubbles generated. The brown precipitate of AuNP-DHLA was purified by washing with toluene and chloroform and all solvents were evaporated. Addition of 5 mL of 0.1 M NaOH caused deprotonation of the COOH groups of the dried AuNP-DHLA and thus rendering the AuNP soluble in the water phase. The gold nanoparticles were purified by passing through the membrane of a 30 KDa molecular weight cut-off (MWCO) centrifuge filter (Millipore) and the particles were concentrated, followed by buffer exchange. [file 1477-3155-11-7-S4.doc]

**S4: Synthesis protocol of AuNP.** In brief, 462.62 mg Didodecyldimethylammonium bromide (DDAB) was dissolved in 10 mL toluene and 86.135 mg decanoic acid was dissolved 5 mL toluene to give stock solution of 100 mM. Gold precursor solution (25 mM) was prepared by dissolving an 6.8 mg of gold (III) chloride (AuCl3) in the in 0.8 mL 100 mM DDAB solution. In a typical synthesis, 1 ml of freshly prepared Tetrabutylammonium borohydride (TBAB) solution (25.73 mg in 1 mL of DDAB solution) was mixed with 0.625 ml decanoic acid stock solution under vigorous stirring and 0.8 ml gold precursor solution was injected leading instantaneously to a dark-red solution of Gold nanoparticles (AuNPs) capped with DDAB. After two hours the solution was centrifuged (2500 rpm, 30 min) to remove free surfactants, reduction agents and smaller nanoparticles. The precipitate of AuNPs was then re-dissolved in 2.5 ml DDAB stock solution.

*Ligand exchange*: To a 2.5 mL solution of AuNP-DDAB freshly reduced 0.104 mg lipoic acid (LA) was added and stirred until no bubbles generated. The brown precipitate of AuNP-DHLA was purified by washing with toluene and chloroform and all solvents were evaporated. Addition of 5 mL of 0.1 M NaOH caused deprotonation of the COOH groups of the dried AuNP-DHLA and thus rendering the AuNP soluble in the water phase. The gold nanoparticles were purified by passing through the membrane of a 30 KDa molecular weight cut-off (MWCO) centrifuge filter (Millipore) and the particles were concentrated, followed by buffer exchange.
