# Supplementary material for: Chemistry of conjugation to gold nanoparticles affects G-protein activity differently
Source: J Nanobiotechnology. 2013 Mar 19;11:7. doi: 10.1186/1477-3155-11-7 (PMC3614441; doi:10.1186/1477-3155-11-7)
Supplement: Additional file 5: Figure S5 — High resolution Transmission electron microscopic (HRTEM) images of AuNP-DHLA. Sample was diluted and directly added on carbon-coated copper TEM grids and the solvent evaporated to form a dry particle film. Images confirm very narrow size distribution. Scale bar corresponds to 50 nm, 20 nm, 5 nm for panel A, B and C respectively. 50 particles were randomly selected and size distribution was measured using Image-J software, resulting in 5.92 nm ± 0.5219 in diameter. [file 1477-3155-11-7-S5.doc]

**
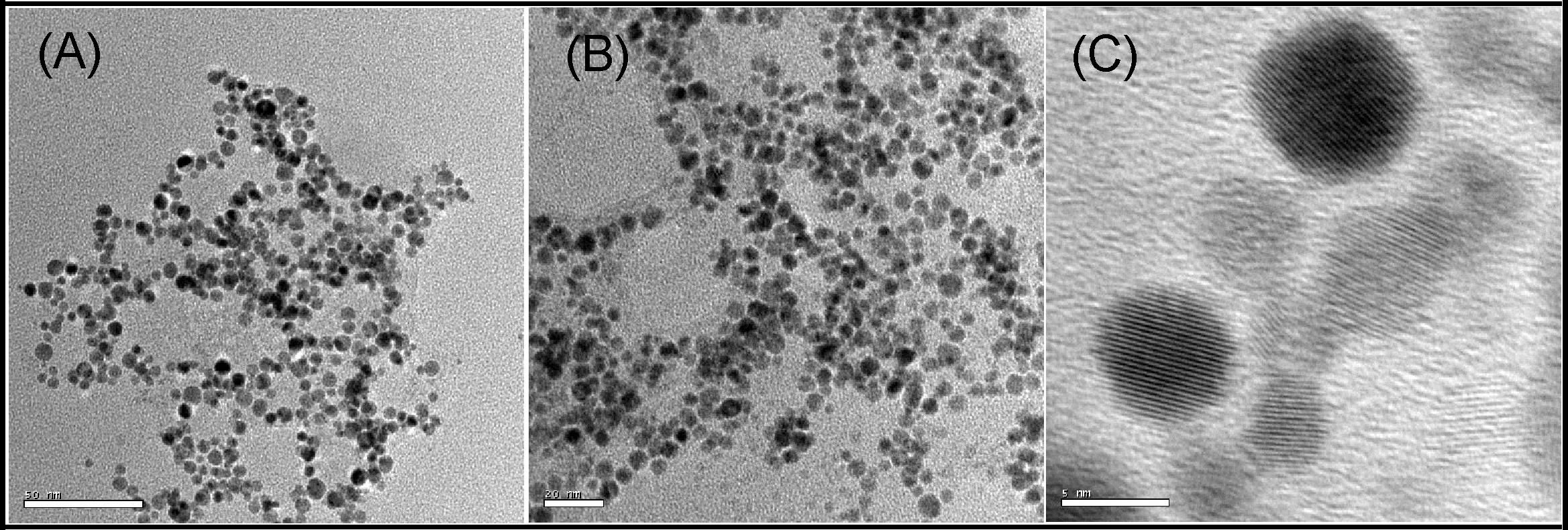
**

**Figure S5: High resolution Transmission electron microscopic (HRTEM) images of AuNP-DHLA.** Sample was diluted and directly added on carbon-coated copper TEM grids and the solvent evaporated to form a dry particle film. Images confirm very narrow size distribution. Scale bar corresponds to 50 nm, 20 nm, 5 nm for panel A, B and C respectively. 50 particles were randomly selected and size distribution was measured using Image-J software, resulting in 5.92 nm ± 0.5219 in diameter.
