# Supplementary material for: Chemistry of conjugation to gold nanoparticles affects G-protein activity differently
Source: J Nanobiotechnology. 2013 Mar 19;11:7. doi: 10.1186/1477-3155-11-7 (PMC3614441; doi:10.1186/1477-3155-11-7)
Supplement: Additional file 6: Figure S6 — Surface plasmon resonance of AuNP. UV-vis absorption spectra of the as-prepared gold nanoparticles (AuNP-DDAB, dotted lines) and after ligand exchange (AuNP-DHLA, solid line). Au samples with DDAB capping were dissolved in toluene, sample with DHLA capping in aqueous solution. AuNP capped with DDAB showed strong plasmon resonance in the range of 520-530 nm. Plasmon resonance was preserved after ligand exchange with DHLA. This confirms stability of AuNP in aqueous medium. [file 1477-3155-11-7-S6.doc]

**
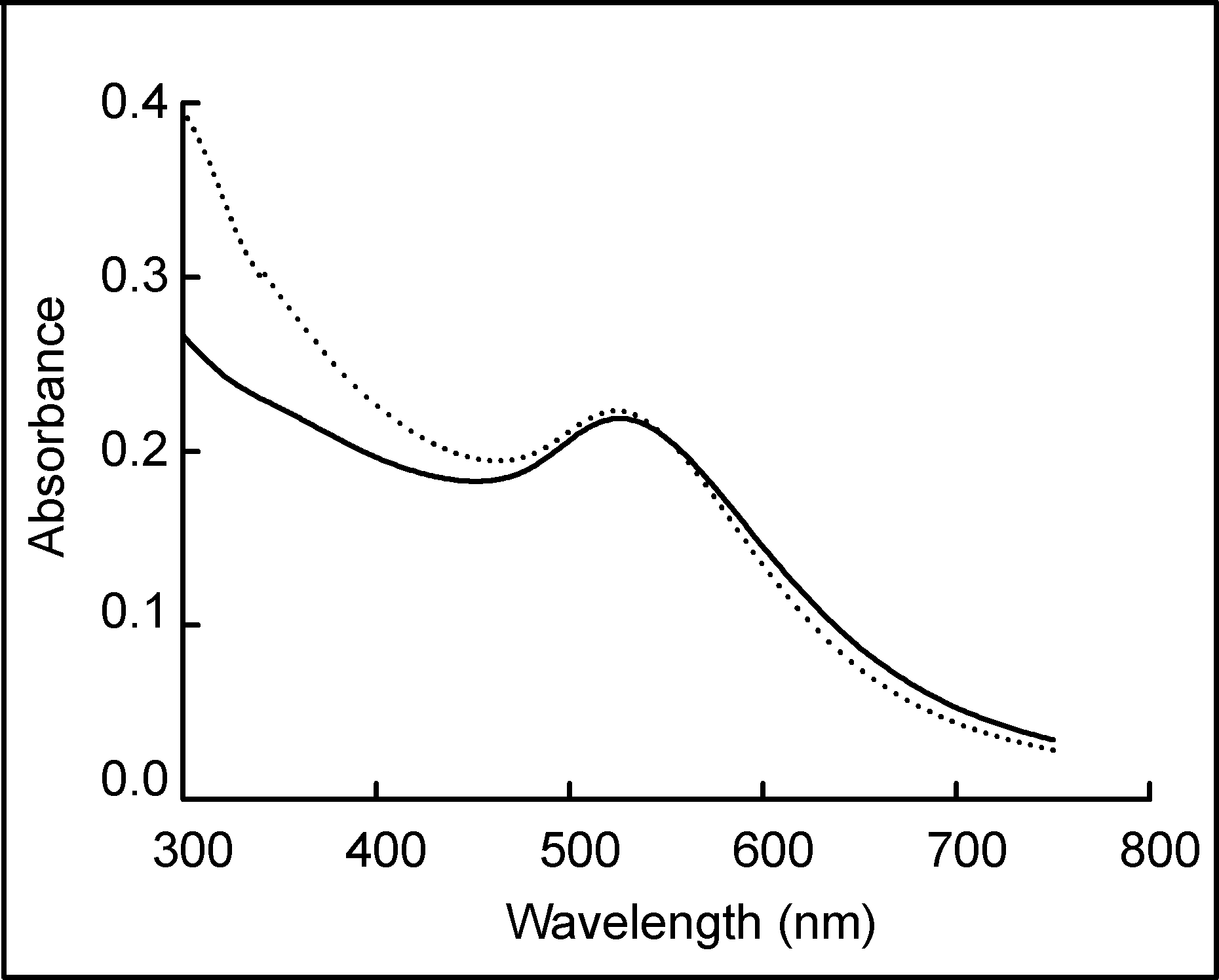
**

**Figure S6: Surface plasmon resonance of AuNP**. UV-vis absorption spectra of the as-prepared gold nanoparticles (AuNP-DDAB, dotted lines) and after ligand exchange (AuNP-DHLA, solid line). Au samples with DDAB capping were dissolved in toluene, sample with DHLA capping in aqueous solution. AuNP capped with DDAB showed strong plasmon resonance in the range of 520-530 nm. Plasmon resonance was preserved after ligand exchange with DHLA. This confirms stability of AuNP in aqueous medium.
