# Supplementary material for: Chemistry of conjugation to gold nanoparticles affects G-protein activity differently
Source: J Nanobiotechnology. 2013 Mar 19;11:7. doi: 10.1186/1477-3155-11-7 (PMC3614441; doi:10.1186/1477-3155-11-7)
Supplement: Additional file 7: S7 — Purification of Gαi1 protein. Cells were grown at 37°C to A600 nm of ~ 0.7 and then induced with 100 μM isopropyl-β-D-thiogalactoside (IPTG). The culture was then grown for 16 hours at 23°C. Cells were harvested by centrifugation, and the resulting pellets were resuspended in a buffer containing 20 mM Tris–HCl (pH 8.0), 300 mM NaCl, 2 mM MgCl2, 10 μM GDP. For purification, the cells were sonicated using an ultrasonicator (Vibracell Sonics and Materials, Inc. Newtown, CT, USA). The lysate was centrifuged at 4°C (45 min at 12,000 rpm). The resulting supernatant was loaded onto a nickel nitrilotriacetic acid (Ni–NTA) Superflow resin column (Qiagen, Hilden, Germany) that was equilibrated with 20 mM Tris–HCl (pH 8.0), 300 mM NaCl, 2 mM MgCl2, 10 μM GDP buffer. The protein loaded resin was washed with 10 column volumes with wash buffer 1 [20 mM Tris–HCl (pH 8.0), 300 mM NaCl, 2 mM MgCl2, 10 mM imidazole] and then with 5 column volume with wash buffer 2 [20 mM Tris–HCl (pH 8.0), 300 mM NaCl, 2 mM MgCl2, 30 mM imidazole]. The bound protein was eluted with 2 column volumes of elution buffer [20 mM Tris–HCl (pH 8.0), 300 mM NaCl, 2 mM MgCl2, 10 μM GDP 300 mM imidazole]., were pooled and concentrated to a volume of 1 mL and loaded onto a Superdex 200 26/60 column (GE Healthcare) that was equilibrated in buffer [5 mM Hepes-Na (pH 8.0), 10 mM NaCl, 0.5 mM MgCl2, 1 μM GDP]. After elution, the protein-containing fractions were pooled, concentrated and stored at −80°C. [file 1477-3155-11-7-S7.doc]

**S7: Purification of Gα_i1_ protein.** Cells were grown at 37°C to A_600 nm_ of ~ 0.7 and then induced with 100 μM isopropyl-β-D-thiogalactoside (IPTG). The culture was then grown for 16 hours at 23°C. Cells were harvested by centrifugation, and the resulting pellets were resuspended in a buffer containing 20 mM Tris–HCl (pH 8.0), 300 mM NaCl, 2 mM MgCl_2_, 10 μM GDP. For purification, the cells were sonicated using an ultrasonicator (Vibracell Sonics and Materials, Inc. Newtown, CT, USA). The lysate was centrifuged at 4°C (45 min at 12,000 rpm). The resulting supernatant was loaded onto a nickel nitrilotriacetic acid (Ni–NTA) Superflow resin column (Qiagen, Hilden, Germany) that was equilibrated with 20 mM Tris–HCl (pH 8.0), 300 mM NaCl, 2 mM MgCl_2_, 10 μM GDP buffer. The protein loaded resin was washed with 10 column volumes with wash buffer 1 [20 mM Tris–HCl (pH 8.0), 300 mM NaCl, 2 mM MgCl_2_, 10 mM imidazole] and then with 5 column volume with wash buffer **2** [20 mM Tris–HCl (pH 8.0), 300 mM NaCl, 2 mM MgCl_2_, 30 mM imidazole]. The bound protein was eluted with 2 column volumes of elution buffer [20 mM Tris–HCl (pH 8.0), 300 mM NaCl, 2 mM MgCl_2_, 10 μM GDP 300 mM imidazole]., were pooled and concentrated to a volume of 1 mL and loaded onto a Superdex 200 26/60 column (GE Healthcare) that was equilibrated in buffer [5 mM Hepes-Na (pH 8.0), 10 mM NaCl, 0.5 mM MgCl_2_, 1 μM GDP]. After elution, the protein-containing fractions were pooled, concentrated and stored at −80° C.
